# First identification of *Anaplasma phagocytophilum *in both a biting tick *Ixodes nipponensis* and a patient in Korea: a case report

**DOI:** 10.1186/s12879-020-05522-5

**Published:** 2020-11-11

**Authors:** Seung Hun Lee, Na-Ri Shin, Choon-Mee Kim, Sungdo Park, Na Ra Yun, Dong-Min Kim, Dong Sik Jung

**Affiliations:** 1 Division of Bacterial Disease Research, Center for Infectious Disease Research, Korea National Institute of Health, Osong, Cheongju-si, 28159 Chungcheongbuk-do Republic of Korea; 2 Premedical Science, College of Medicine, Chosun University, Gwangju, Republic of Korea; 3 Department of Internal Medicine, College of Medicine, Chosun University, 588 Seosuk-dong, Dong-gu, Gwangju, 61453 Republic of Korea; 4 Department of Internal Medicine, College of Medicine, Dong-A University, Busan, Republic of Korea

**Keywords:** Human granulocytic anaplasmosis, Tick bites, *Anaplasma phagocytophilum*, *Ixodes nipponensis*

## Abstract

**Background:**

Human granulocytic anaplasmosis (HGA) is a tick-borne infectious disease caused by *Anaplasma phagocytophilum*. To date, there have been no reported cases of *A. phagocytophilum* infection found in both the biting tick and the patient following a tick bite.

**Case presentation:**

An 81-year-old woman presented with fever following a tick bite, with the tick still intact on her body. The patient was diagnosed with HGA. The tick was identified as *Ixodes nipponensis* by morphological and molecular biological detection methods targeting the 16S rRNA gene. The patient’s blood was cultured after inoculation into the human promyelocytic leukemia cell line HL-60. *A. phagocytophilum* growth was confirmed via culture and isolation. *A. phagocytophilum* was identified in both the tick and the patient’s blood by *Anaplasma*-specific *groEL-* and *ankA-*based nested polymerase chain reaction followed by sequencing. Moreover, a four-fold elevation in antibodies was observed in the patient’s blood.

**Conclusion:**

We report a case of a patient diagnosed with HGA following admission for fever due to a tick bite. *A. phagocytophilum* was identified in both the tick and the patient, and *A. phagocytophilum* was successfully cultured. The present study suggests the need to investigate the possible incrimination of *I. nipponensis* as a vector for HGA in Korea.

**Supplementary information:**

**Supplementary information** accompanies this paper at 10.1186/s12879-020-05522-5.

## Background

Human granulocytic anaplasmosis (HGA) is a tick-borne infectious disease caused by *Anaplasma phagocytophilum*, an obligate intracellular bacterium, which grows in membrane-bound vacuoles of humans and animals [1]. The annual incidence of this disease was reported to be 6.3 cases per million person-years between 2008 and 2012 in the United States [2]. *Ixodes scapularis*, a vector for Lyme disease or babesiosis, is known to be the primary vector of HGA in the United States, whereas *Ixodes pacificus* (western black-legged tick) and *Ixodes ricinus* (castor bean tick) are the presumed vectors across the western United States and Europe, respectively [3, 4]. In Korea, following the first report of anaplasmosis in 2013, HGA has been described as an emerging infectious disease [5]. However, to date, no studies have investigated the vectors of HGA in Korea.

We therefore performed molecular detection and isolation of *A. phagocytophilum* from the blood of a patient who presented with fever after a tick bite, with the tick still attached to the body.

## Case presentation

### Case

An 81-year-old woman was hospitalized with a chief complaint of fever. She developed fever, headache, and vomiting 5 days prior to admission and was treated conservatively at a local hospital. Three days prior to admission, her fever symptoms recurred and were accompanied by several vomiting episodes, abdominal pain, and shortness of breath.

**Fig. 1:**
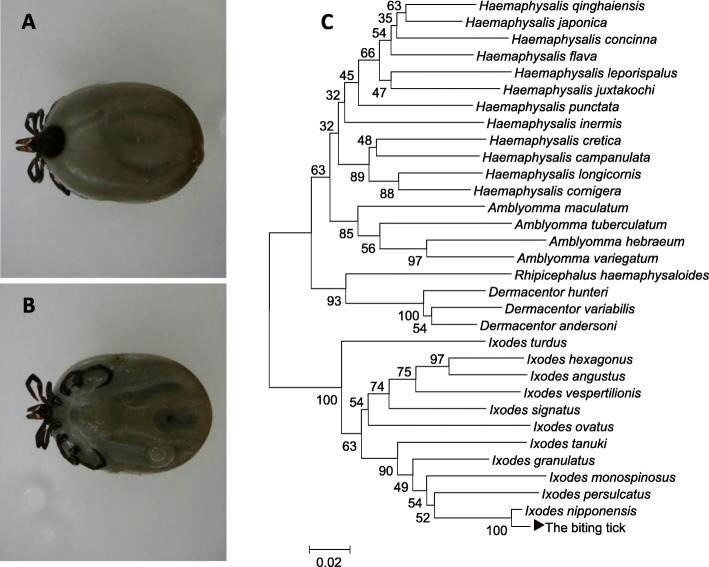
Gross findings of the tick removed from the right neck area of the patient. Dorsal view (**a**) and ventral view (**b**) of the tick. Identification of *Ixodes nipponensis* from the phylogenetic tree analysis based on the 16S rRNA gene of the tick of 367-bp amplicons produced from the conventional polymerase chain reaction (**c**)

**Fig. 2:**
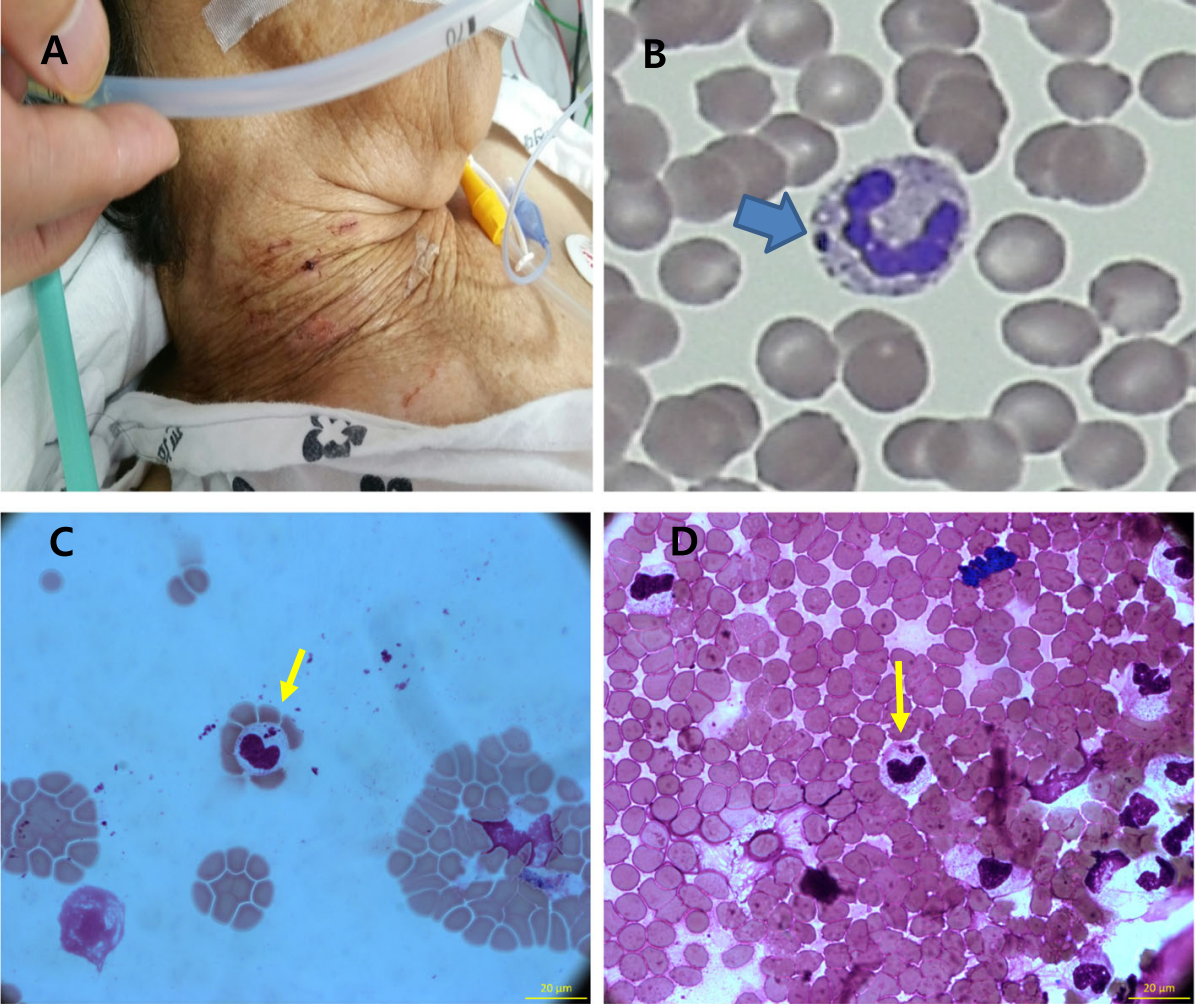
Site of the tick bite (right neck area) (**a**). Suspected morulae in the peripheral blood smear (thick arrow) (**b**). Diff-Quik stained-peripheral blood smear showing neutrophils with intracellular inclusions (narrow arrows). Original magnification, 1000x (**c**, **d**)

Consequently, the patient visited the emergency room of a local hospital and was misdiagnosed with cholecystitis, based on the results of abdominal computed tomography, and tenderness in her right upper quadrant on examination. She underwent laparoscopic cholecystectomy the next day and received antibiotic therapy; however, her fever persisted. Her guardian identified a mass-like lesion on the right side of her neck where an adhesive patch had been applied and notified the medical staff. Upon confirming the presence of a tick, the tick was removed (May 30). A single dose of 100 mg doxycycline was administered, and the patient (along with the tick) (Fig. 1a) was transferred to the Chosun University Hospital in Gwangju City, Korea (Fig. 2a). On admission (May 30), physical examination indicated that the patient was febrile, with a blood pressure of 90/60 mmHg and body temperature of 37.8 °C. She looked acutely ill on inspection, and her pulse and respiratory rates were 131 beats/min and 22 breaths/min, respectively. Skin examination confirmed a tick bite lesion on the right side of the neck (Fig. 2). Laboratory investigations further revealed the following findings: white blood cell count, 9,260/mm^3^ (91.6% polymorphonuclear leukocytes); hemoglobin level, 12.6 g/dL; platelet count, 24,000/mm^3^; aspartate aminotransferase level, 71.1 U/L; alanine aminotransferase level, 38.7 U/L; and creatine phosphokinase level, 255 U/L.

The patient indicated that she was a resident of a rural area and lived close to a cattle farm with approximately 100 cows; the farm was separated from her house by only a single wall. The patient had a vegetable garden at her home, where she spent a considerable amount of time working almost every day both in the morning and evening. During the medical history interview on admission, the patient reported that she first noted discomfort in her right side of neck on April 22, 2017, which was prior to the initial appearance of symptoms. When her husband was asked to inspect the area, he mentioned that it appeared to be a wart. At that time, she developed a papular lesion, accompanied by itching and persistent discomfort, for which she began applying adhesive patches on May 6, 2017.

We suspected that the patient was bitten by the tick at least 1 or 2 days prior to showing the site to her husband on April 22. *A. phagocytophilum* was identified from the patient specimen through polymerase chain reaction (PCR). Subsequently, the patient was treated with 100 mg doxycycline twice daily for 10 days. The patient showed improvement in the C-reactive protein level and pancytopenia, normalization of liver function test results, and symptom improvement with treatment. She was discharged accordingly.

### Tick identification

The developmental stage and species of the tick were morphologically identified by microscopy, using standard taxonomic keys (https://shire.science.uq.edu.au/parasites/arachnids/ticks/ticks-identification.php). After morphological identification, the tick was washed in 70% ethanol for 2–5 times, washed again in sterilized distilled water for 3 times, and dried by placing on a sterile filter paper. The tick was then placed in the MK28 hard tissue grinding tube (Bertin Technologies, Rockville, MD, USA), 600 μL of phosphate-buffered saline (10% fetal bovine serum, 5% penicillin/streptomycin) was added, and ground by using FastPrep®-24 Classic Instrument (MP Biomedicals, Solon, OH, USA). The genomic DNA of the tick was then extracted, using a G-spin Total DNA Extraction Kit (iNtRON Biotechnology, Seongnam, Korea), following the manufacturer’s protocol. Total RNA was isolated using the Viral Gene-spin™ Viral DNA/RNA Extraction Kit (iNtRON Biotechnology, Seongnam, Korea). For molecular biological identification of the tick, genomic DNA was subjected to the mitochondrial 16S rRNA gene-targeted PCR assay [6].

### PCR detection for vector-borne infectious disease pathogens

PCR was performed on the tick lysate and the patient’s blood to detect for vector-borne infectious pathogens. DNA was extracted from the patient’s initial blood specimen and from the tick, using the QIAamp Blood and Tissue Mini Kit (Qiagen, Hilden, Germany), following the manufacturer’s instructions [7]. *Anaplasma*-specific *ankA*- and *groEL*-based nested PCRs (n-PCRs) were performed; the primers used for *groEL* were HS1/HS6 (for the first PCR) and HS43/HS45 (for the nPCR)*,* while those for *ankA* were ANK-F1/ANK-R1 (for the first PCR) and ANK-F2/ANK-R2 (for the nPCR) [8, 9]. To detect *Rickettsia* spp.*, Rickettsia*-specific *ompA* fragment was amplified using primers R190.70F/RR190.701R (for the first PCR) and R190.70F /RR190.602R (for the N-PCR) [10, 12]. For the detection of *Borrelia* spp., *pyrG* N-PCR was performed by amplifying the CTP synthase genes, using primers pyrG-1F/pyrG-1R (for the first PCR) and pyrG-2F/pyrG-2R (for the N-PCR) [13]. The gene encoding the *Orientia tsutsugamushi* 56-kDa antigen was amplified using the designed primers 56BO-144F/56BO-1395R (for the first PCR) and 56BO-406F/56BO-1088R (for the N-PCR). To assess for severe fever with thrombocytopenia syndrome virus RNA, we performed reverse transcription PCR, as previously described [11]. The genomic DNAs of *A. phagocytophilum* KZ_A3, *Rickettsia conorii, Borrelia burgdorferi,* and the *O. tsutsugamushi* Karp strain served as positive controls for *Anaplasma*-specific, *Rickettsia*-specific, *Borrelia*-specific and *O. tsutsugamushi*-specific targets, respectively. In each PCR run, the reaction mixture without template DNA served as the negative control. All primer sequences and PCR cycling conditions are shown in Table 1.

**Table 1:** Conditions and primers used for PCR, amplified product base pair size, and references used for PCR in this study

PCR assay	Name of primer seq (5′-3′)	PCR conditions				Product Size (bp)	Reference
		Denaturation	Annealing	Extension	Cycles		
		(°C/sec)	(°C/sec)	(°C/sec)			
16 s rRNA PCR for tick identification	16S - 1-F (CTGCTCAATGAATATTTAAATTGC)	95/45	55/60	72/90	40	450	(https://shire.science.uq.edu.au/parasites/arachnids/ticks/ticks-identification.php)
	16S-1-R (CGGTCTAAACTCAGATCATGTAGG)						
*ankA* N-PCR for *Anaplasma phagocytophilum*	ANK-F1 (GAAGAAATTACAACTCCTGAAG)	95/30	53/30	72/60	35	705	[7]
	ANK-R1 (CAGCCAGATGCAGTAACGTG)						
	ANK-F2 (TTGACCGCTGAAGCACTAAC)	95/30	52/30	72/60	5	664	
	ANK-R2 (ACCATTTGCTTCTTGAGGAG)	95/30	54/30	72/60	25		
*groEL* N-PCR for *Anaplasma phagocytophilum*	HS1 TGGGCTGGTA(A/C)TGAAAT	94/60	48/120	70/90	3	1300	[8]
	HS6 CCICCIGGIACIA(C/T)ACCTTC	88/60	52/120	70/90	37		
	HS43 AT(A/T)GC(A/T)AA(G/A)GAAGCATAGTC	94/60	48/120	70/90	3	528	
	HS45 ACTTCACG(C/T)(C/T)TCATAGAC	88/60	55/120	70/90	37		
*ompA* N-PCR for *Rickettsia* spp.	R190.70F (ATGGCGAATATTTCTCCAAAAA)	94/30	50/30	72/60	40	634	[9]
	RR190.701R (GTTCCGTTAATGGCAGCATCT)						
	R190.70F (ATGGCGAATATTTCTCCAAAAA)	94/30	51/30	72/30	5	535	
	RR190.602R (AGTGCAGCATTCGCTCCCCCT)	94/30	55/30	72/30	30		
56 kDa N-PCR for *Orientia tsutsugamushi*	56BO-144F (YGYAGAATCTRCTCGCTTGG)	94/60	60/60	72/60	35	1250	In this study
	56BO-1395R (agctaMccctRcaccaaBac)						
	56BO-406F (CCWCCTCARCCTACTAtrTGC)	94/30	61/30	72/45	30	680	
	56BO-1088R (gcWgctgctRctgcttcttg)						
*pyrG* N-PCR for *Borrelia* spp.	pyrG-1F (ATTGCAAGTTCTGAGAATA)	94/20	45/30	72/30	30	801	[10]
	pyrg-1R (CAAACATTACGAGCAAATTC)						
	pyrG-2F (GATATGGAAAATATTTTATTTATTG)	95/30	45/30	72/30	5	707	
	pyrg-2R (AAACCAAGACAAATTCCAAG)	95/30	47/30	72/30	5		
		95/30	49/30	72/30	25		
RT-PCR for SFTSV	MF3 (GATGAGATGGTCCATGCTGATTCT)	95/20	58/40	72/30	35	560	[11]
	MR2 (CTCATGGGGTGGAATGTCCTCAC)						

Positive PCR products from the patient’s blood and the tick were sequenced and aligned to the GenBank database to identify revealed bacterial agents at the species level and to compare our sequences with published sequences using BLAST analysis and ClustalW alignment (www.clustal.org).

### Phylogenetic analysis

DNA sequences were identified and analyzed using DNASTAR Lasergene v6 (DNASTAR, Madison, WI, USA). A phylogenetic tree was constructed by the neighbor-joining method using ClustalX version 2.0 (www.clustal.org/), based on sequences of the amplified *Anaplasma*-specific *groEL* and *ankA* gene fragments from the specimen and from GenBank. A bootstrap analysis was performed using 1000 replicates to improve the confidence level of the phylogenetic tree.

### Peripheral blood smear

A thick peripheral blood smear was examined for the presence of *A. phagocytophilum* in the neutrophils. A drop of the patient’s blood was smeared onto a glass slide, air-dried, and stained with Diff-Quik solution (Sysmex Corporation, Kobe, Japan), following the standard procedure. The smear was observed under a microscope at a magnification of 1000x.

### *A. phagocytophilum* culture and isolation

For *A. phagocytophilum* culture and isolation, the human promyelocytic leukemia cell line HL-60 (KCLB-10240) was maintained in RPMI1640 medium (Gibco, Thermo Fisher Scientific, MA USA), supplemented with 2% fetal bovine serum (Gibco, Thermo Fisher Scientific, MA USA) and 2 mM L-glutamine at 37 °C and under 5% CO_2_ conditions. The buffy coat or tick lysate solution was inoculated into HL-60 cells. Infected cells were cultured under the same conditions as above, with regular medium changes (cell density of 1–5 × 10^5^ cells/mL). They were then stained with Diff-Quik at 2–3-day intervals and were subsequently cytocentrifuged for microscopic examination.

### Indirect immunofluorescence antibody assay

An indirect immunofluorescence assay (IFA) was performed, following the standard procedure and/or manufacturer’s instructions (Fuller Laboratories, Fullerton, CA, USA) [14]. In-house IFA commenced with antigen fixation of the heavily-infected HL-60 cells onto Teflon-coated slides with acetone. An anti-*A. phagocytophilum* serum (Fuller Laboratories, Fullerton, CA, USA) was then added to the antigen slides, and the slides were incubated in a humidity chamber at 37 °C for 30 min. DyLight 488-labeled goat anti-human IgG or IgM (Fuller Laboratories, Fullerton, CA, USA) was used as a secondary antibody under the same conditions. The antigen slides were counterstained with 0.005% Evans blue and mounted for fluorescence microscopy. Manufacturer’s slides containing infected HL-60 cells (Fuller Laboratories, Fullerton, CA, USA) and the human serum were used as positive and negative controls, respectively. For the serological diagnosis of scrub typhus, murine typhus, and Lyme disease, IgM and IgG antibodies against the standard *O. tsutsugamushi* antigen (Gilliam, Karp, Kato, and Boryong strains), *Rickettsia typhi*, and *B. burgdorferi* were assessed using in-house IFA [15].

### Ultrastructural analysis of A. phagocytophilum

To investigate *A. phagocytophilum* infection, the cytoplasmic area of occupied vacuoles was explored by electron microscopy at the ultrastructural level. The cultured cells were stained with Diff-Quik at 2–3-day intervals, cytocentrifuged to confirm the infection level, and then examined, using scanning and transmission electron microscopy (SEM and TEM, respectively).

## Results

### Tick identification

Morphological analysis confirmed that the tick was a female adult *I. nipponensis* (Fig. 1a, b). This was further confirmed by molecular identification and phylogenetic tree analysis of the 367-bp 16S rRNA amplicons produced using conventional PCR (C-PCR) followed by sequencing (Fig. 1c).

### *A. phagocytophilum* detection

**Fig. 3:**
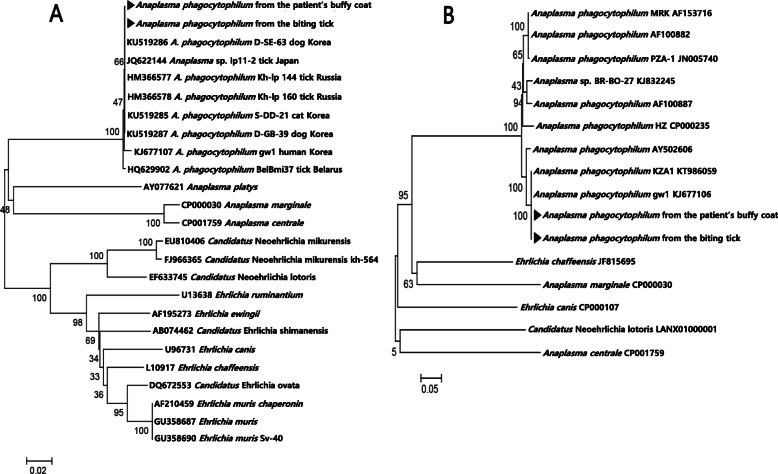
Phylogenetic tree analysis, following *groEL* and *ankA* gene-targeted PCR of the patient’s buffy coat at the time of admission and of the tick (**a**, *groEL*; **b**, *ankA*)

On initial peripheral blood smear analysis, the Wright–Giemsa-stain showed findings suggestive of morulae (Fig. 2b), whereas Diff-Quik confirmed this by revealing intracytoplasmic inclusion bodies in the neutrophils (Fig. 2c, d). The n-PCR was performed on the patient’s buffy coat and the tick by using the 347-bp amplicons of *A. phagocytophilum*-specific primers targeting *groEL*. Sequence alignment indicated 100% homology between the patient and the tick (NCBI accession no. MH492313, MH492314), which in turn shared 99% homology with *A. phagocytophilum* isolates D-SE-63 (accession no. KU519286) and S-DD-21 (accession no. KU519285), previously identified in dogs and cats, respectively, in Korea. Sequences were cut to a size of 335-bp for the phylogenetic tree analysis (Fig. 3a) and were shown to form clusters with *A. phagocytophilum* strain D-SE-63 (accession no. KU519286), identified in dogs in Korea, *Anaplasma* spp. (accession no. JQ622144), identified in a Japanese tick, and *A. phagocytophilum* strain Kh-Ip144 (accession no. HM366577), identified in a Russian tick (66 bootstraps).

In terms of the *ankA* gene sequence, sequence alignment indicated 100% homology between the patient and the tick (NCBI accession no. MH492315, MH492316), which also shared 100% homology with *A. phagocytophilum* isolates KZA1 (accession no. KT986059) and gw1 (accession no. KJ677106). Sequences were cut to 837-bp for the phylogenetic tree analysis (Fig. 3b) and further confirmed cluster formation with *A. phagocytophilum* KZA1 (accession no. KT986059) and gw1 (accession no. KJ677106), originally identified in Korean patients (100 bootstraps).

### *A. phagocytophilum* culture using the Patient’s blood

Cytoplasmic inclusions in the infected cells were observed 32 days post-inoculation of HL-60 cells with *A. phagocytophilum* isolated from the patient’s buffy coat (Supplementary Figure 1A–D). IFA involving both IgM and IgG antibodies from the antiserum positively reacted with *A. phagocytophilum,* which have propagated in the HL-60 cells, with fluorescent morulae filled with bacteria observed surrounding the cell’s cytoplasmic membrane (Supplementary Figure 1E, F). The nPCR and direct sequencing of the isolated bacteria provided positive results and confirmed the presence of the same *A. phagocytophilum* bacterial strain identified in the patient’s blood (named *A. phagocytophilum* KZ_A3).

### *A. phagocytophilum* ultra-structural studies

**Fig. 4:**
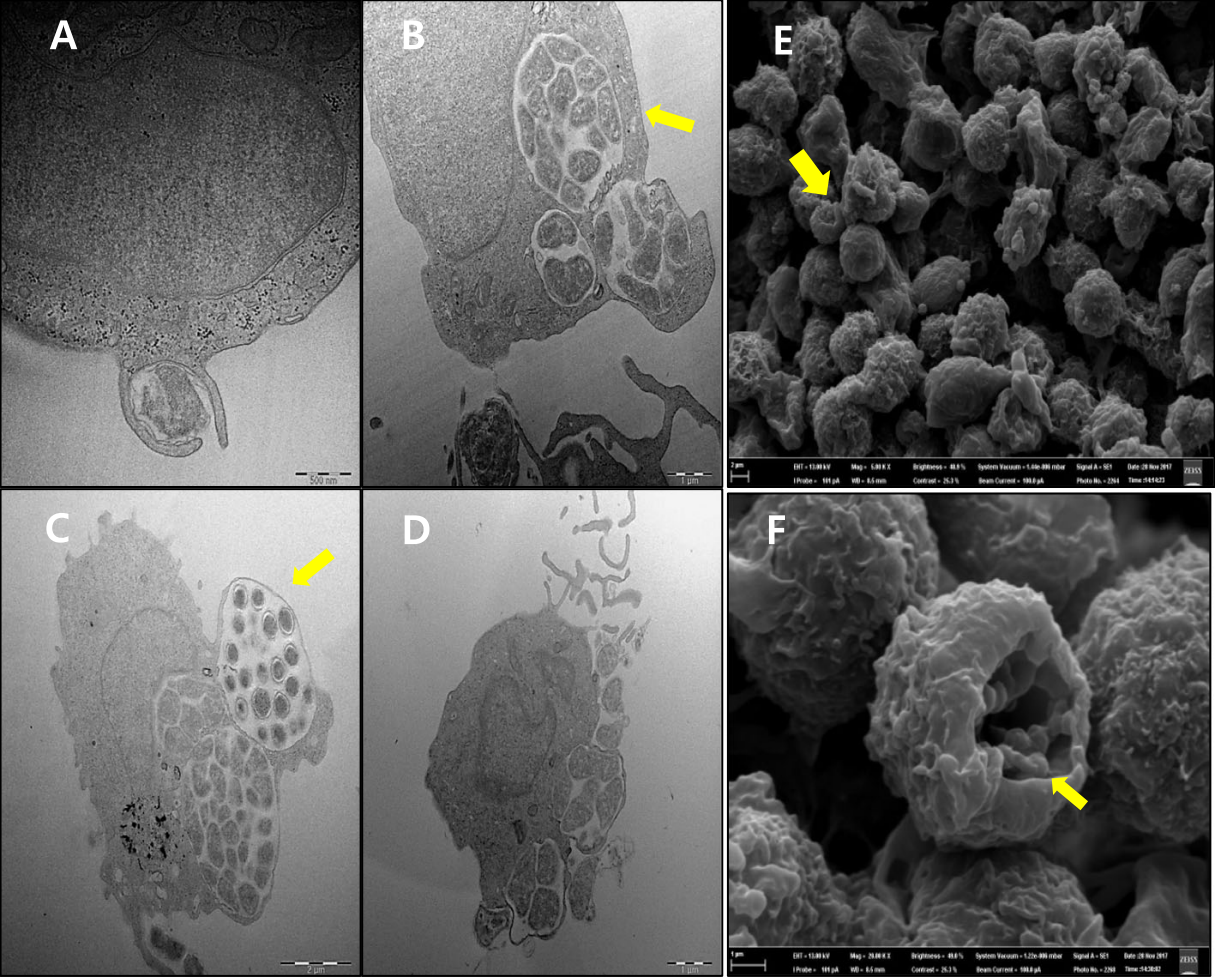
Transmission electron microscopy showing the infection stages of isolated *A. phagocytophilum* KZ_A3-infected HL-60 cells. Adhesion, replication, invasion, and release stages of the isolated *A. phagocytophilum* within HL-60 cells (**a**-**d**). Representative images of dense-cored cells and reticulate cells; the large arrow indicates a dense nucleoid and a ruffled outer membrane, and the spheroid indicates individual dense-cored cells surrounded by a membranous projection (**c**). Scanning electron micrograph of a cluster of isolated *A. phagocytophilum* KZ_A3 within HL-60 cells (**e**, **f**) *A. phagocytophilum* KZ_A3 were observed to replicate in a large vesicle inside the cell. Here, the vesicle has ruptured, revealing *A. phagocytophilum* (**c**–**d**, 20000x)

The infection stages adhesion, replication, invasion, and release of isolated *A. phagocytophilum* KZ_A3 were visualized within HL-60 cells using TEM (Fig. 4a). Reticulate cells were characterized by dispersed nucleoids (Fig. 4b), a smoother outer membrane than that observed with dense-cored cells (Fig. 4c), and pleomorphism (Fig. 4a–d). Infected cells were further examined by SEM at the ultrastructure level (Fig. 4e, f), which showed the replication of *A. phagocytophilum* KZ_A3 within large vesicles of the cell, with a grape-like cluster appearance. Ruptured vesicles were also observed, revealing a cluster of *A. phagocytophilum* (Fig. 4f). Infectious dense-cored cells were predominantly coccoid bacteria at the late stages of infection (Fig. 4e, f).

### *A. phagocytophilum* culture with tick lysates

HL-60 cell culture at 10 days post-inoculation with *Anaplasma* spp. isolated from the tick lysate showed unexpected microbial growth, which was later observed in other cell culture assays (Supplementary Figure 2). The intracellular morphology was distinct from that of *Anaplasma* spp.*,* and was similar to that of *Rickettsia* spp. With Diff-Quik staining, the unexpected bacteria replicated faster than *Anaplasma* spp.; however, *Anaplasma* spp. was detected in infected cells using in-house IFA, and fluorescent bacteria were observed following incubation with an *A. phagocytophilum* antiserum. This prompted a re-evaluation of the infection by this agent (Supplementary Figure 2).

### PCR studies for other targets

PCR with *Rickettsia* genus-specific primers of 435-bp amplicons targeting the outer membrane protein A (*ompA*) was performed on the tick. Sequence alignment indicated a 100% identity to the *R. monacensis* strain MT34 (accession no. JX972178) isolated from a Korean tick. We confirmed that the unexpected intracellular bacteria, detected during tick lysate culturing, were *R. monacensis*. Although *Anaplasma* spp. was detected during tick lysate culture, its isolation was unsuccessful, possibly due to coinfection. *O. tsutsugamushi, R. typhi,* and *Borrelia* spp. were not found in the tick specimen.

### Confirmation of infection using IFA studies

A 4-fold or greater rise in titer between acute and convalescent sera represented evidence for infection. IFA results of the patient serum showed that both IgM and IgG antibodies to *A. phagocytophilum* were negative at the time of admission (5 days after symptom onset). The levels of IgG (1:160) and IgM (1:64) antibodies increased 10 days after symptoms onset; similarly, the levels of antibodies to *O. tsutsugamushi* and *R. typhi* were also elevated. However, when the patient’s admission blood specimen was used for *ompA*-targeted PCR to detect *R. typhi* and *R. monacensis* and for PCR to detect *O. tsutsugamushi* and *Borrelia* spp., all results were negative (Table 2).

**Table 2:** PCR and antibody test results of the patient

Sample collection day	PCR				IFA							
	***ankA*** nPCR ***groEL*** nPCR	***ompA***	56-kDa nPCR	SFTS C-PCR	***Orientia tsutsugamushi***		***Rickettsia*** ***typhi***		***Anaplasma phagocytophilum***		***Borrelia burgdorferi***	
					IgG	IgM	IgG	IgM	IgG	IgM	IgG	IgM
2017-05-30	**+**	–	–	–	1:2048	< 1:16	1:64	< 1:16	< 1:80	< 1:16	< 1:16	< 1:16
2017-06-02					1:1024	< 1:16	1:64	< 1:16				
2017-06-05	–			–					1:160	1:64	1:16	< 1:16
2017-06-07					1:512	< 1:16	1:64	< 1:16				
2017-06-20	–			–	1:256	< 1:16	1:512	< 1:16	1:640	1:64	1:32	< 1:16
2017-06-26	–			–	1:512	< 1:16	1:512	< 1:16	1:640	1:256	1:16	< 1:16
2017-07-03					1:512	1:16	1:512	< 1:16	1:640	1:64	1:16 WB+	1:16 WB+
2017-07-20					1:512	< 1:16	1:512	< 1:16	1:640	1:512	1:32 WB+	1:16 WB+
2017-08-04					1:512	< 1:16	1:512	< 1:16	1:640	1:256	1:16 WB+	1:16 WB+

*PCR* polymerase chain reaction; *IFA* indirect immunofluorescence assay; *nPCR* nested PCR; *SFTS* severe fever with thrombocytopenia syndrome; *C-PCR* conventional PCR; *WB* western blot (Positive means more than 7 antigen bands were detected using the Microgen diagnostic kit)

## Discussion and conclusions

*A. phagocytophilum* is transmitted by *I. scapularis* in New England and the north-central United States, *I. pacificus* in the western United States, *I. ricinus* in Europe, and *I. persulcatus* in Asia [16]. In Japan, which is in close proximity to Korea, *A. phagocytophilum* has been identified in *I. persulcatus* and *I. ovatus* and was first reported in 2005 [17]. Furthermore, 4.6% of *I. persulcatus* isolated from northern China was reported to have tested positive for *A. phagocytophilum* [18]. Kim et al. [19] reported that among 1467 ticks (1463 *Haemaphysalis longicornis*, 3 *I. persulcatus*, and 1 *I. turdus*) collected from 9 Korean provinces, 35 *H. longicornis* and 1 *I. persulcatus* were confirmed to be positive for *A. phagocytophilum*, whereas 1 *I. persulcatus* tested positive for *Ehrlichia chaffeensis*.

In a molecular detection study of *Anaplasma* spp. infection rates in ticks collected from migratory birds on Hongdo Island, Korea, *A. phagocytophilum* was detected in only 1 *I. nipponensis* nymph, among a total of 212 ticks (40 *Haemaphysalis flava*, 12 *H. longicornis*, 146 *I. turdus*, 13 *I. nipponensis*, and 1 *I. ornithophilia*) [20]. Thus, it is speculated that *H. longicornis*, *I. persulcatus,* and *I. nipponensis* may act as anaplasmosis vectors in Korea. *A. phagocytophilum* has previously been identified in three tick species (*H. longicornis, I. persulcatus,* and *I. nipponensis*) in Korea, but no direct transmission from ticks to humans has been confirmed.

Since the first report on the serological and molecular detection of HGA in Korea in 2002, numerous studies on *A. phagocytophilum* vectors have been performed [21] However, there are no reported cases that have characterized the species of the biting tick or that have detected the presence of an identical *A. phagocytophilum* strain in both the biting tick and the patient, particularly with a tick that was intact on the patient’s body. In the present study, the identical *A. phagocytophilum* strain was identified in not only the patient who was diagnosed with HGA following admission for fever symptoms after a tick bite but also in the tick itself. Moreover, *A. phagocytophilum* was successfully cultured from both the patient’s blood and the biting tick,

Follow-up antibody test results of the tick DNA sample showed elevated antibodies not only for *A. phagocytophilum* but also for *O. tsutsugamushi*, *R. typhi*, *B. burgdorferi* and *R. monacensis* *monacensis*. Therefore, the possibility of coinfection or sequential infection could not be dismissed.

In our previous study on 317 patients with scrub typhus, 96.2% showed elevated IgM antibodies (1:10) within 1 month of symptom onset, whereas 67.4% continued to test positive for IgM antibodies at the 6-month follow-up test [22]. A recent infection would have correlated with elevated levels of IgM antibodies; however, this was not observed in the present case. Moreover, all PCR results on the patient’s blood and the tick were negative for *O. tsutsugamushi*, *R. typhi*, and *B. burgdorferi.* Thus, we believed that the probability of a past infection, sequential infection, or antibody cross-reactivity may be higher than that of a coinfection. Similarly, the cross-reactivity between *E. canis* and *A. phagocytophilum* has been reported previously in experimental animal [23]. However, additional studies are needed specifically to clarify the cross-reactivity between *Anaplasmosis* spp. and *B. burgdorferi*.

The studies showed that the full engorgement of ticks require 7 to12 days of attachment on the host before shedding off to continue with their life cycle. However, it has also been suggested that some ticks may require 30 days or longer [5]. Nonetheless, the literature has shown that the incubation period for human anaplasmosis ranges from 5 to 21 days [24].

A limitation of this study was that the analyzed tick specimens were completely engorged by the blood of the infected woman, and the positivity to *A. phagocytophilum* observed in the DNA of the tick specimen could have come from the patient’s blood and not from the salivary glands of the tick. Therefore, we could not confirm that *I. nipponensis* is a vector for *A. phagocytophilum*. Furthermore, since the patient came from an area with a high risk of tick bites, we could not eliminate the likelihood of undetectable tick bites at other sites of her body.

## Conclusion

In conclusion, we presented a case involving a patient diagnosed with HGA following admission for fever due to a tick bite. An identical *A. phagocytophilum* strain was identified in both the patient and the tick, and *A. phagocytophilum* was successfully cultured from the patient’s blood. Nevertheless, further studies are warranted to determine whether *I. nipponensis* can act as a possible vector for anaplasmosis in Korea.

## Supplementary information

**Additional file 1 Supplement 1.** Morulae of *Anaplasma phagocytophilum.* Light micrograph of *A. phagocytophilum* cultured in a human promyelocytic cell line (A: dpi32, B: dpi37, C: dpi39, D: Cell passage 2). Diff-Quik staining (A–D). The arrow indicates *A. phagocytophilum* KZ_A3. Original magnification (A-D; 400x) of in-house immunofluorescence staining of isolated *A. phagocytophilum* from the patient within the human promyelocytic cell line (dpi 37). Culture preparations stained by IFA using an anti-*A. phagocytophilum* serum. The arrow indicates intracytoplasmic inclusions filled with numerous bacteria. Fluorescence magnification (E–F; 400x). IFA of *A. phagocytophilum* KZ_A3 in infected HL-60 cells (37 dpi). The cells were treated in turn, with antiserum and anti-human IgG (E) or IgM (F) conjugate to detect *A. phagocytophilum*. The yellow arrows indicate intracytoplasmic inclusions filled with numerous bacteria (400x magnification). **Supplement 2.** Morulae, suspected of *A. phagocytophilum*. Light micrograph of *A. phagocytophilum* cultured in a human promyelocytic cell line, using the tick lysate (A: dpi10, B: dpi10). Diff-Quik staining (A, B). The arrow indicates *A. phagocytophilum* KZ_A3. Original magnification (A; 400x, B; 1000x) of in-house immunofluorescence staining of the *A. phagocytophilum* infected tick lysate solution within the human promyelocytic cell line (day 10). Culture preparations were stained by IFA using an anti-*A. phagocytophilum* serum. The arrow indicates intracytoplasmic inclusions filled with numerous bacteria. Fluorescence magnification (C–D; 400x).

**Additional file 2.** The same description has been provided as a word file, as per the journal requirements.

## Data Availability

The datasets analyzed during the current study are available at National Center for Biotechnology Information (NCBI) repository. (Accession numbers; MH492313, MH492314, MH492315, MH492316, KU519285, KU519286, JQ622144, HM366577, KT986059, KJ677106, KT986059, KJ677106, JX972178).

## Abbreviations

HGA: Human granulocytic anaplasmosis
PCR: Polymerase chain reaction
IFA: Indirect immunofluorescent assay
SEM: Scanning electron microscopy
TEM: Transmission electron microscopy
