# Supplementary figures and images for: First identification of Anaplasma phagocytophilum in both a biting tick Ixodes nipponensis and a patient in Korea: a case report
Source: BMC Infect Dis. 2020 Nov 11;20:826. doi: 10.1186/s12879-020-05522-5 (PMC7656494; doi:10.1186/s12879-020-05522-5)

## Slide 1
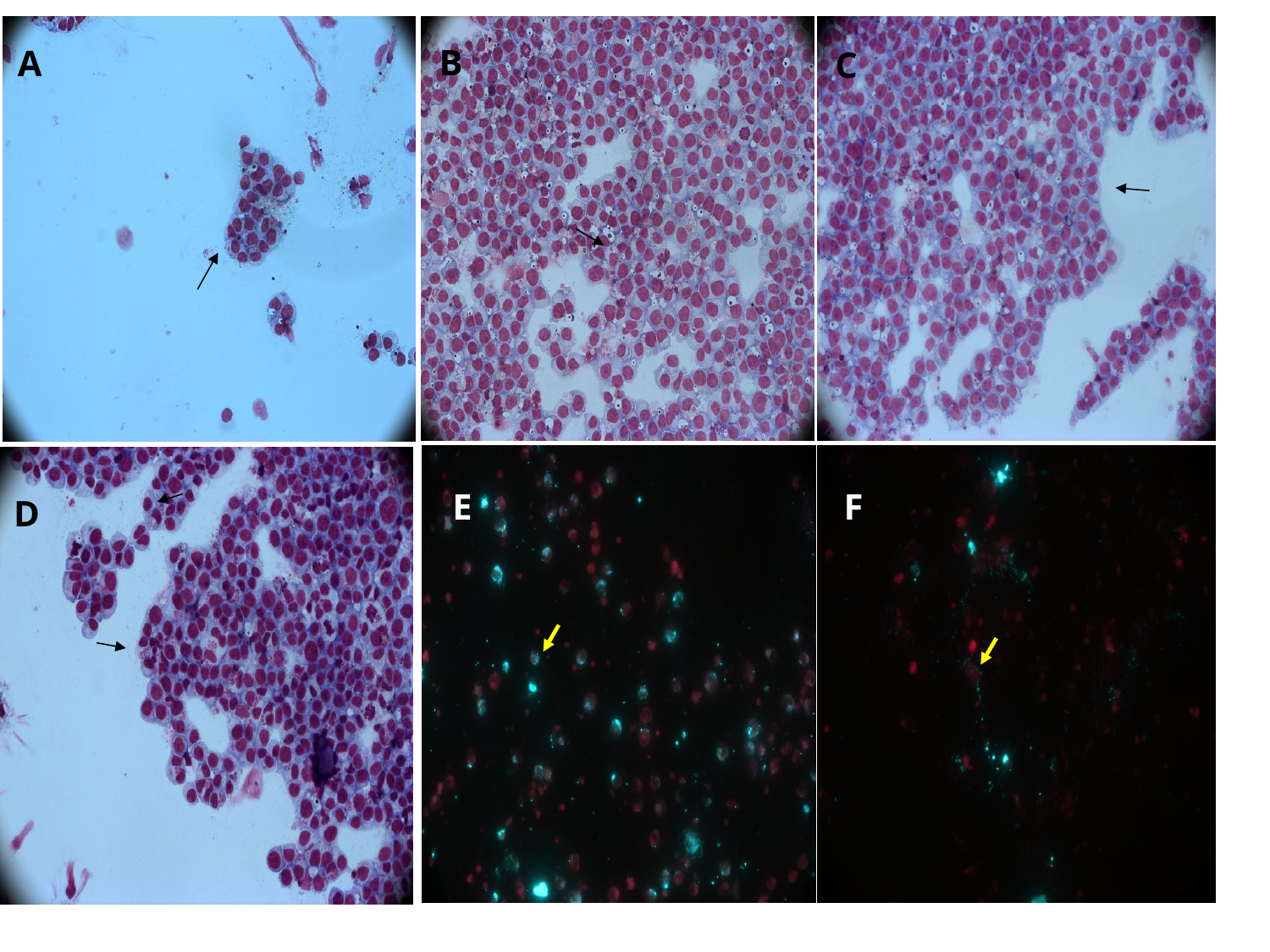

A
B
C
E
F
D

## Slide 2
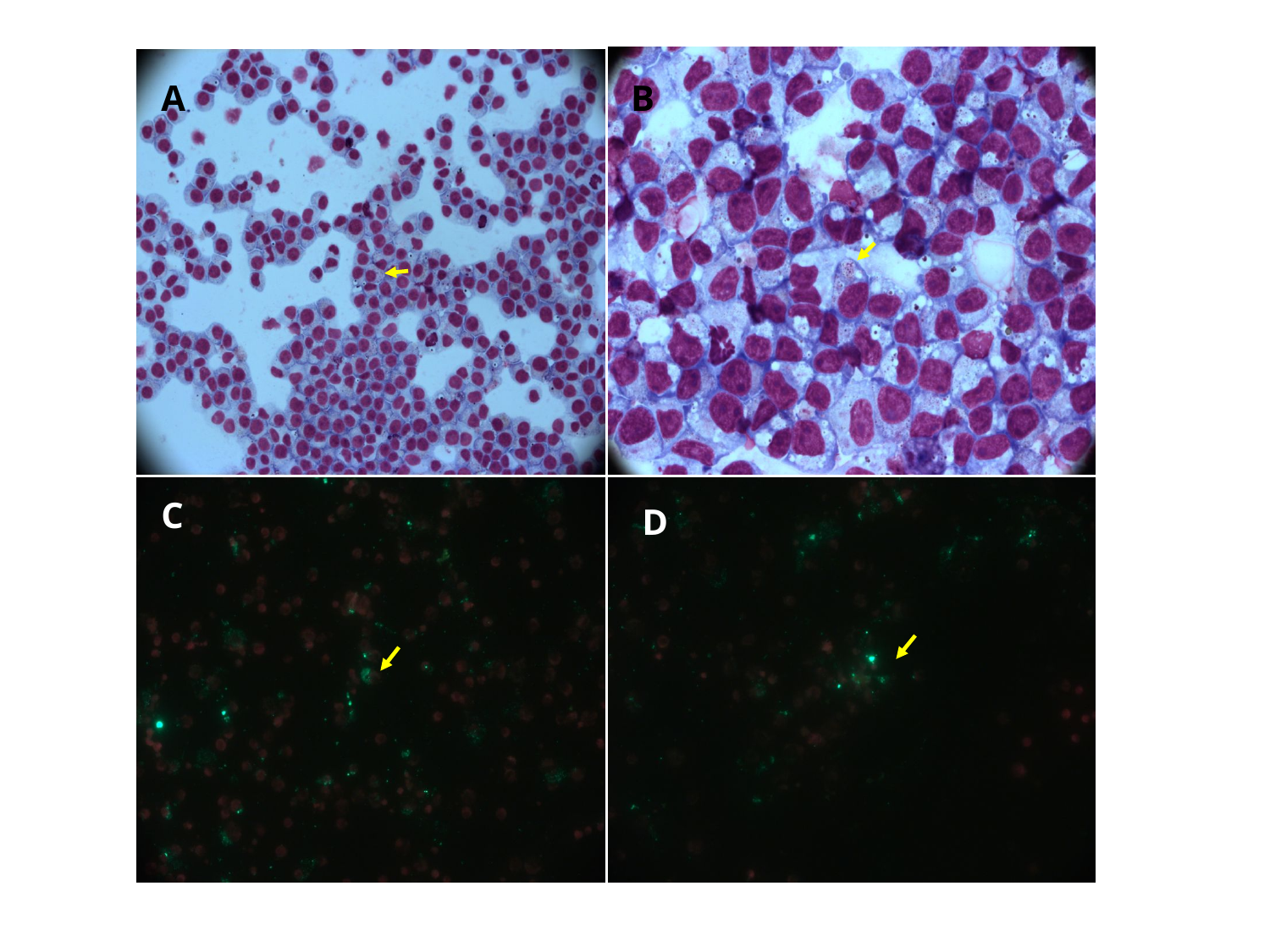

B
A
C
D

Supplement: Supplementary file 1 — Additional file 1 Supplement 1. Morulae of Anaplasma phagocytophilum. Light micrograph of A. phagocytophilum cultured in a human promyelocytic cell line (A: dpi32, B: dpi37, C: dpi39, D: Cell passage 2). Diff-Quik staining (A–D). The arrow indicates A. phagocytophilum KZ_A3. Original magnification (A-D; 400x) of in-house immunofluorescence staining of isolated A. phagocytophilum from the patient within the human promyelocytic cell line (dpi 37). Culture preparations stained by IFA using an anti-A. phagocytophilum serum. The arrow indicates intracytoplasmic inclusions filled with numerous bacteria. Fluorescence magnification (E–F; 400x). IFA of A. phagocytophilum KZ_A3 in infected HL-60 cells (37 dpi). The cells were treated in turn, with antiserum and anti-human IgG (E) or IgM (F) conjugate to detect A. phagocytophilum. The yellow arrows indicate intracytoplasmic inclusions filled with numerous bacteria (400x magnification). Supplement 2. Morulae, suspected of A. phagocytophilum. Light micrograph of A. phagocytophilum cultured in a human promyelocytic cell line, using the tick lysate (A: dpi10, B: dpi10). Diff-Quik staining (A, B). The arrow indicates A. phagocytophilum KZ_A3. Original magnification (A; 400x, B; 1000x) of in-house immunofluorescence staining of the A. phagocytophilum infected tick lysate solution within the human promyelocytic cell line (day 10). Culture preparations were stained by IFA using an anti-A. phagocytophilum serum. The arrow indicates intracytoplasmic inclusions filled with numerous bacteria. Fluorescence magnification (C–D; 400x). [file 12879_2020_5522_MOESM1_ESM.pptx]
