# Supplementary material for: First identification of Anaplasma phagocytophilum in both a biting tick Ixodes nipponensis and a patient in Korea: a case report
Source: BMC Infect Dis. 2020 Nov 11;20:826. doi: 10.1186/s12879-020-05522-5 (PMC7656494; doi:10.1186/s12879-020-05522-5)
Supplement: Supplementary file 2 — Additional file 2. The same description has been provided as a word file, as per the journal requirements. [file 12879_2020_5522_MOESM2_ESM.docx]

**Supplementary Figure 1.** Morulae of *Anaplasma phagocytophilum*. Light micrograph of *A. phagocytophilum* cultured in a human promyelocytic leukemia cell line (A: 32 days postinoculation (dpi), B: 37 dpi, C: 39 dpi, D: cell passage 2). Diff-Quik staining (A–D). The arrow indicates *A. phagocytophilum* KZ_A3. Original magnification (A–D, ×400). In-house immunofluorescence staining of isolated *A. phagocytophilum* within the human promyelocytic leukemia cell line (37 dpi). Culture preparations stained by indirect immunofluorescent antibody assay using an anti-*A. phagocytophilum* serum. The arrow indicates intracytoplasmic inclusions filled with numerous bacteria. Fluorescence magnification (E–F, ×400).

**Supplementary Figure 2.** Light micrograph of *Anaplasma phagocytophilum* cultured in a human promyelocytic leukemia cell line using the tick lysate solution (A: 10 days postinoculation (dpi), B: 10 dpi). Diff-Quik staining (A, B). The arrow indicates *A. phagocytophilum* KZ_A3. Original magnification (A, ×400; B, ×1,000). In-house immunofluorescence staining (C, D) of *A. phagocytophilum*-infected tick lysate solution within a human promyelocytic leukemia cell line (day 10). Culture preparations stained by indirect immunofluorescent antibody assay using an anti-*A. phagocytophilum* serum. The arrow indicates intracytoplasmic inclusions filled with numerous bacteria. Fluorescence magnification (C–D, ×400)
